# Redefining universal health coverage in the age of global health security

**DOI:** 10.1136/bmjgh-2016-000255

**Published:** 2017-03-07

**Authors:** Vageesh Jain, Azeem Alam

**Affiliations:** King's College London, School of Medicine, London, UK

**Keywords:** Health policies and all other topics, Infections, diseases, disorders, injuries

**Summary box**

- Redefining the scope of universal health coverage (UHC) can provide benefits for global health security.
- UHC may affect how a country is able to prevent, detect or respond to an infectious disease outbreak, but its impact is difficult to measure and depends greatly on the definition of UHC.
- Future initiatives to move towards UHC must include methods of measuring the national availability, quality and distribution of public health services, particularly epidemiological surveillance, and research and development to further integrate global health security and health system strengthening.

## Introduction

Universal health coverage (UHC) has received a great deal of attention over the past decade, with the WHO spearheading the global advocacy effort. Studies have demonstrated the ability of UHC to reduce mortality, and overcome existing health inequalities to create more equitable systems.^1^ ^2^

However, the role of UHC in preventing, detecting and responding to disease outbreaks as per International Health Regulations (IHR), particularly during public health emergencies of international concern (PHEIC), is less clear. Past epidemics, including Ebola or H1N1 influenza, have not provided the opportunity to assess the impact that UHC may have on global health security. In the Ebola outbreak, the virus was largely limited to the West African region, among countries that all had poorly functioning health systems. The H1N1 virus proved to be too feeble to allow an analysis of how resilient different health systems were (based on UHC), in combating the 2009 pandemic.

UHC, in its existing form, has the potential to improve global health security through various mechanisms. First, low financial barriers can stimulate demand for health services and facilitate early case detection,^3^ one of the foremost factors in dictating the course of an outbreak. Second, UHC may protect people from financial catastrophe. High healthcare expenditures push people into poverty, further increasing their long-term risk of ill health, particularly through communicable disease. Third, UHC protects against economic downturn, with unemployment associated with a lower mortality in UHC countries compared with those without.^2^ This is important in the context of epidemics, as seen in the recent Ebola outbreak, where the World Bank estimates $1.6 billion were foregone in GDP in 2015, due to the economic impact of the outbreak.^4^ Aside from the clinical and economic benefits, there also lies a societal benefit in creating a more equitable and just system of health whereby the poor do not bear a disproportionate burden of disease.

The high death toll inflicted by Ebola has reinvigorated health system strengthening efforts, to which UHC is fundamental.^5^ ^6^ But what exactly should UHC involve, to improve global health security? The ongoing Zika virus outbreak, raging through the Americas, provides an opportunity to examine the complex relationship between UHC and infectious disease control.

### UHC and the Zika outbreak

Table 1 shows the number of confirmed Zika cases in Latin America, as well as incidence rates (calculated as suspected and confirmed cases/100 000 population) by country, as of 9 November 2016. UHC and non-UHC countries have been included for comparison. UHC was defined as legislation mandating universal healthcare, along with >90% of the population with effective financial protection, and >90% skilled birth attendance. The definition used here reflects that used in the WHO first global symposium on health systems research, to measure the global prevalence of UHC.^7^ An exception was made for the Venezuelan health system, which, despite fulfilling our criteria, has been in a state of collapse since 2015. Pre-Zika birth rates and UN human development index rankings were also included, to assess whether these key socioeconomic factors can help to explain national Zika burden.

**Table 1:** Zika incidence rates and key socioeconomic indicators, by country^8–10^

Country	2014 birth rate (per 1000 women)	UN human development index ranking	Zika incidence rate (cases per 100 000)
Andean area			
Guyana	19	124	3776.5
Colombia*	16	97	216.0
Venezuela	20	71	193.5
Brazil*	15	75	148.0
Ecuador*	21	88	21.5
Bolivia	24	119	7.8
Peru	20	84	0.4
Southern cone			
Paraguay	21	112	8.3
Argentina*	18	40	4.2
Chile*	13	42	0
Uruguay*	14	52	0
Latin Caribbean			
St Martin	16	75	7972.2
Guadeloupe	–	57	6614.4
Puerto Rico	10	61	889.2
Dominican Republic	21	101	48.9
Haiti	25	163	27.3
Cuba*	10	67	0.03
Central America			
Honduras	21	131	390.8
El Salvador	17	116	185.0
Belize	23	101	159.7
Costa Rica*	15	69	80.1
Panama	19	60	62.9
Nicaragua	20	125	33.1
Guatemala	27	128	19.5
Mexico*	19	74	4.7

*Universal Health Coverage.

**Figure 1:**
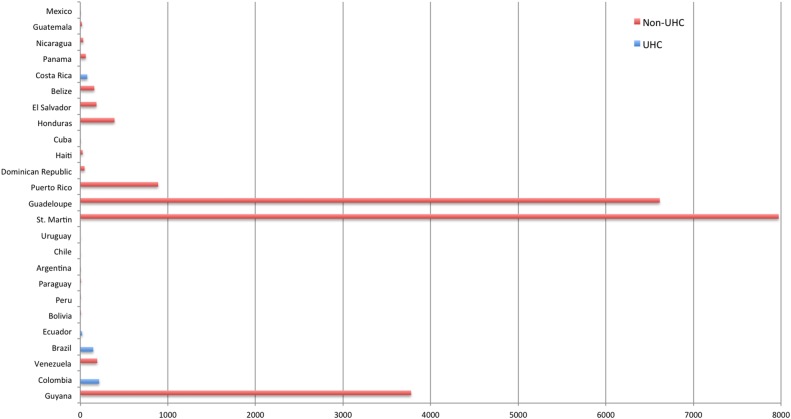
Zika incidence rates per 100 000 population in Latin American countries.^8^

When looking specifically at the health systems of Latin American countries, figure 1 shows that areas with the highest incidence of Zika are in non-UHC countries. This does not provide evidence, however, that UHC countries such as Brazil are better equipped to overcome Zika. Table 1 shows that neighbouring non-UHC countries, for instance, Bolivia and Peru, despite having lower UN HDI rankings, and higher pre-Zika birth rates, have lower rates of Zika virus compared with Brazil. In part, this seemingly complex pattern may be due to the importance of other factors such as the nature of the virus, its geographical evolution during this epidemic and various ecological factors regarding mosquito life cycle, prevalence and survival. It is also true that numbers of confirmed cases apparent in UHC countries such as Brazil may in part be a result of more accurate case reporting, due to wider access to, and availability of, health services.

Theoretically, UHC should provide an advantage in the prevention, detection and response to public health emergencies. In practice, the relationship between health policy and epidemic control is more complex, and difficult to measure. The clear lack of a consistent relationship between UHC status and Zika burden does pose an important question; are current efforts to move towards UHC appropriate in the context of public health emergencies?

### Rethinking UHC for global health security

The concept of UHC encompasses three key dimensions; who has access, which services are provided and what proportion of the cost is covered.^11^ Traditionally, much focus has been on day-to-day functions of a health system, and the proportion of the population with access to affordable healthcare.

From a global health security standpoint, it is imperative to further dissect the ‘which services?’ component of UHC. Specifically, the view must not be to excessively focus on secondary and tertiary level services, for instance, hospital maternity units, in the case of Zika. To engage with emerging or re-emerging disease, sufficient attention must be paid to national public health services, epidemiological surveillance and research and development (R&D).

Brazil has a system of UHC, where specialised outpatient and hospital services continue to receive the vast majority of healthcare investment, receiving on average, more than six times the funding for epidemiological surveillance during the period 2004–2011.^12^ The regional variance in these investment figures may have played a role in the course and focus of the Zika epidemic. In Pernambuco, the state with the highest reported rates of microcephaly, more than 10 times as much was being spent on specialist hospital services compared with epidemiological surveillance.^12^ It is widely recognised that where surveillance and response activities are well funded and implemented, the disease and economic impacts of infectious diseases are better controlled.^13^ Governments looking to develop sustainable capacity to respond to rapidly spreading epidemics must recognise often overlooked public health services as vital, and adequately finance them as part of the UHC model. The superfluous focus on tertiary and urgent care, at the expense of public health functions, holds true not just for Brazil but exists in many countries, including the UK.^14^

There are currently over 30 active projects worldwide, pursued by the public and private sector, aiming to develop a vaccine against Zika.^15^ The WHO recently developed a target product profile for Zika vaccines, which provides an outline of the desired characteristics for a vaccine. But reaching this point alone has taken months, due to a limited understanding of viral epidemiology as well as the importance of various routes of transmission. Adequate investment in R&D is paramount in effectively responding to mysterious emerging pathogens and drug-resistant infections. National health policy dialogue on implementing effective, high-quality systems of UHC must therefore not be bereft of R&D investment considerations. Including R&D under the framework of broader health policy may also lead to research investment decisions aligning with existing epidemiological evidence, particularly important in countries facing the dual burden of disease.^16^

With many countries now implementing plans to achieve UHC, and the increasing threat of emerging pathogens^17^ as well as antimicrobial resistance,^18^ it is essential to highlight the vital role public health and R&D services have in discussions around UHC.

### Measuring UHC

The WHO proposes eight core health service coverage indicators to measure UHC. These include reproductive health measures (family planning, antenatal care, skilled birth attendance), immunisation (three doses of diphtheria, tetanus and pertussis-containing vaccine), infectious disease (antiretroviral therapy, tuberculosis treatment) and improved water sources/sanitation. Nevertheless, national policy on UHC and health systems varies enormously across the world. With so many ways to measure it, when can a nation declare the achievement of UHC? For instance, take Venezuela, one of the Latin American countries affected by Zika, using the skilled birth attendance indicator, the country would have UHC. However, if DPT immunisation coverage (more reflective of public health capability) were used instead, it would not be considered a UHC country.^19^ A universally agreed measurement of UHC, through a robust set of available indicators, is crucial considering the dynamic nature of political will, and volatility of cyclical agendas.

From a public health emergency perspective, there is a case to include a wider set of indicators in measuring UHC, enabling a more thorough measurement of national capabilities in public health, especially epidemiological surveillance. Additional health system resilience measures could include national emergency preparedness plans, systems for medical countermeasures in emergencies, reporting networks/protocols and laboratory capacity. Critics may point out that measuring the capacity of such areas in resource-poor settings is not as important as assessing the capacity of local hospital services. But many of the aforementioned public health functions serve to develop and support existing tertiary services. In addition, it would be hazardous to suggest countries have achieved UHC without developing sustainable capacity for the key public health services that are integral to infectious disease prevention, detection and response.

## Conclusions

Much of the discourse surrounding UHC does not sufficiently acknowledge the vital role public health and R&D services have in advancing global health security. The current narrative on UHC must expand to include measuring the availability, quality and distribution of such services, and encourage investment in them as part of the move towards UHC. The intersection between global health security and UHC must be highlighted and further scrutinised in an era of emerging pathogens, globalisation and a need for the rapid development of health system resilience.
